# Archives of Electrology and Neurology

**Published:** 1874-06

**Authors:** 


### Bibliographical.

---

ARCHIVES OF ELECTROLOGY AND NEUROLOGY, *a Journal of Electro-Therapeutics and Nervous Diseases.* Edited by George M. Beard, A.M., M.D. Subscription price, $2.50 a year; single numbers, $1.50

The aim will be to make the Archives of Electrology and Neurology both scientific and practical. Each number will contain not merely long essays that appeal only to a limited body of specialists, but rather a variety of *short* articles some of which will be of general interest. The plan of the Journal will be as follows:

1. Papers on important questions in Electrology, including Electro-Physics, Electro-Physiology, Electro-Pathology and Electro-Therapeutics—medical and surgical.

2. Reports of interesting and suggestive cases treated by electricity, including operations by electrolysis and galvano-cautery.

3. Papers on important questions in Neurology, including Neoro-Physiology, Neuro-Pathology and the Diagnosis and Therpeutics of Diseases of the Nervous System.

4. Papers on important questions in Psychology, Insanity, Mental Hygiene and Medical Jurisprudence.

5. Descriptions of new and improved electric apparatus for medical use.

6. Abstracts of papers published in home and foreign journals.

7. Reviews of books.

8. Miscellaneous notes.

It is the desire of the editor that the Archives may be distinguished by candor and fairness, that its impartiality may be absolute, and that, especially in the reports of cases, the love of truth may be always conspicuous.

Already a large number of the highest living authorities in the general and special branches of electrology and neurology have promised to contribute to this journal. In this list of actual and prospective contributors are the names of Althaus, of London; Benedict, of Vienna; Hitzig and Eulenberg, of Berlin; Erb, of Heidelberg; Vater, of Prague; Onimus and Tripier, of Paris; and in this country nearly all the best known writers on these themes, not only in New York and Brooklyn, but in all the great medical centers.

It is expected that specialists in Ophthalmology, in Otology, in Laryngology, in Neuro-Physiology and Pathology, in Gynæcology, in Psychology, in Medical Jurisprudence, in Dermatology, in Syphilography and in Sanitary Science, as well as in the general departments of Electrology and Neurology, will treat of the subjects in which they have obtained mastership; and that general practitioners in medicine and surgery will give their experience in those departments where their opportunities for observation are superior to those of specialists.

This Journal will, therefore, not be exclusively the organ of specialists in Electro-Therapeutics and Nervous diseases. It is designed for specialists in all departments, and for the general practitioner in city and country; and a fair proportion of the articles in every number will be prepared with reference to the wants of those who are beginning the study of Electrology and Neurology, who feel the need of a knowledge of the elements of those sciences, and who, therefore, require to be addressed in plain language and on practical themes.

Subscriptions may be sent to the publisher, Thos. L. Clacher, 107 East Twenty-eighth street, New York.

